# webCEMiTool: Co-expression Modular Analysis Made Easy

**DOI:** 10.3389/fgene.2019.00146

**Published:** 2019-03-06

**Authors:** Lucas E. Cardozo, Pedro S. T. Russo, Bruno Gomes-Correia, Mariana Araujo-Pereira, Gonzalo Sepúlveda-Hermosilla, Vinicius Maracaja-Coutinho, Helder I. Nakaya

**Affiliations:** ^1^ Department of Clinical and Toxicological Analyses, School of Pharmaceutical Sciences, University of São Paulo, São Paulo, Brazil; ^2^ Advanced Center for Chronic Diseases–ACCDiS, Facultad de Ciencias Químicas y Farmacéuticas, Universidad de Chile, Santiago, Chile; ^3^ Centro de Genómica y Bioinformática, Facultad de Ciencias, Universidad Mayor, Santiago, Chile

**Keywords:** co-expression analysis, systems biology, transcriptomics, web tool, data integration

## Abstract

Co-expression analysis has been widely used to elucidate the functional architecture of genes under different biological processes. Such analysis, however, requires substantial knowledge about programming languages and/or bioinformatics skills. We present webCEMiTool,^1^ a unique online tool that performs comprehensive modular analyses in a fully automated manner. The webCEMiTool not only identifies co-expression gene modules but also performs several functional analyses on them. In addition, webCEMiTool integrates transcriptomic data with interactome information (i.e., protein-protein interactions) and identifies potential hubs on each network. The tool generates user-friendly html reports that allow users to search for specific genes in each module, as well as check if a module contains genes overrepresented in specific pathways or altered in a specific sample phenotype. We used webCEMiTool to perform a modular analysis of single-cell RNA-seq data of human cells infected with either Zika virus or dengue virus.

## Introduction

Cellular processes are driven by multiple interacting molecules whose activity level must be dynamically regulated (Kitano, 2002). As a result, genes belonging to the same signaling and metabolic pathway or sharing similar functions will tend to be co-expressed across conditions (Wang et al., 2016). Co-expression gene module analysis creates networks comprising sets of genes (i.e., modules) whose expression is highly correlated. Such analysis was applied to reveal functional modules related to infectious (Janova et al., 2015), inflammatory (Beins et al., 2016), and neurological (Voineagu et al., 2011) diseases, as well as several types of cancer (Sharma et al., 2017).

Weighted gene co-expression network analysis (WGCNA) is a widely used method to identify co-expressed gene modules (Zhang and Horvath, 2005). In order to run WGCNA, however, users are required to be familiar to programming environments, as well as to manually select parameters. These features prevent researchers with insufficient knowledge of R to identify gene modules from transcriptome data sets.

Based on our Bioconductor R package named CEMiTool (Russo et al., 2018), we developed a user-friendly web-based application that allows scientists with no background in bioinformatics to perform comprehensive co-expression network analysis.

## Materials and Methods

The web interface of webCEMiTool was developed to allow users to quickly generate comprehensive analyses without the need of installing any specific program or internet browser. The only requirement for running the modular analysis is a data set containing the expression levels of all genes in samples under different biological conditions (herein defined as “classes”). There is no defined range number of samples but our previous study suggests a minimum of 15 samples per data set (Russo et al., 2018). Although it was primarily designed for transcriptome data (i.e., RNA-seq or microarrays), it can also be potentially used for identifying modules of proteins, cytokines, and even metabolites. webCEMiTool will then automatically select the input genes and identify the co-expression modules. Each module contains a set of genes whose expression follows a similar pattern.

**Figure 1:**
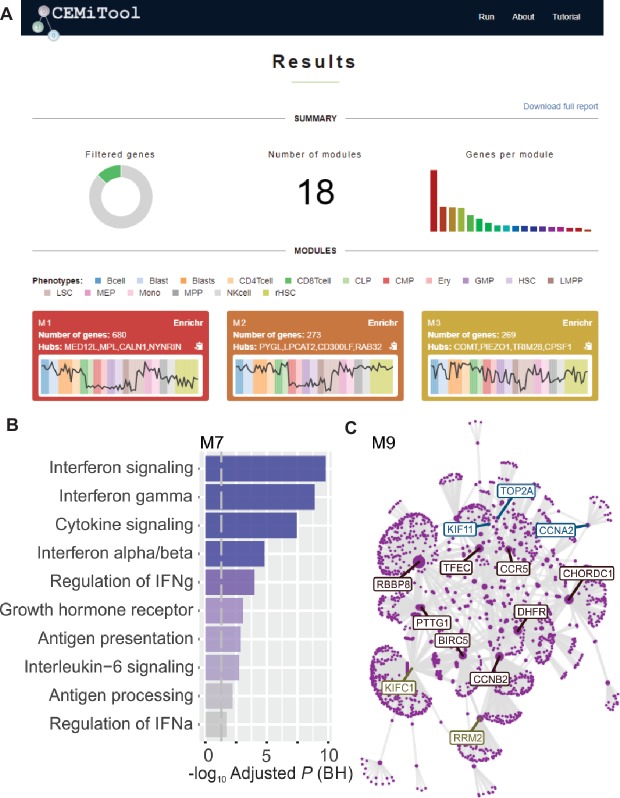
webCEMiTool overview. **(A)** webCEMiTool results summary – The donut chart represents the proportion of selected genes by the unsupervised filter. The front page also displays the number of modules obtained, as well as a bar chart depicting the number of genes in each module. Module profile plots illustrate the median expression activity of genes from the modules across each sample. The colors represent the different sample classes. **(B)** Overrepresentation analysis – This depicts the −log10 adjusted p-value (Benjamini-Hochberg) of the enriched pathways in a module (pathways defined by user-inputted .gmt file). **(C)** Gene network of a module – The top most connected genes (hubs) are labeled and colored based on whether they were originally present in the module (blue), or inserted from a user-inputted interaction file (red), or both (green).

We implemented, within webCEMiTool, a feature that assesses the activity of gene modules on each class of samples. For this, the users only have to provide a sample annotation tab-delimited text file that informs the class of each sample. A “profile plot” showing the median level of individual genes within the module is then displayed in the “Results” section of the tool (Figure 1A).

To enable functional analysis, the users can also check if the gene modules are associated with specific signaling or metabolic pathways (Figure 1B). These pathways can easily be extracted from databases, such as KEGG, Reactome, and MySigDB. Finally, users can integrate the results with interactome data (i.e., protein-protein interactions, transcription factors and their transcribed genes, or even miRNAs and their target genes). This feature enables users to identify critical regulators of modules (Figure 1C), providing valuable insights for experimental validation or potential targets for drugs. Additional details on how to obtain the optional files can be found in the “Tutorial” page of the website.^2^

To demonstrate that our method is robust, we performed an unprecedented large-scale modular analysis with over 1,000 publicly available RNA-seq and microarray data sets and new RNA-seq data of patients infected with Leishmania using the CEMiTool R package version (Russo et al., 2018). Although webCEMiTool and the package have distinct visualization features and are based on different platforms, the core co-expression functionality is essentially the same. The online tool we are describing here is built to enable easy access to gene modular analyses for non-programming researchers, while the R library version is geared towards users with greater knowledge of the R programming language. Additionally, the results dashboard is composed of interactive charts that facilitate interpretation. Moreover, taking advantage of the rising ecosystem of bioinformatics web services, our tool establishes an interface with the Enrichr platform (Chen et al., 2013), enabling a richer experience for our users.

## Results

We demonstrated that webCEMiTool can be applied to analyze expression data at the single cell level. Publicly available viscRNA-Seq data (virus-including single cell RNA-Seq) were obtained from NCBI GEO database (accession number GSE110496) and used as input for the analysis. The data refer to the transcriptome of individual human hepatoma (Huh7) cells, which were infected with either dengue virus (DENV) or Zika virus (ZIKV), using multiplicity of infection (MOI) 0, 1, or 10 (Zanini et al., 2018). Cells collected on four different time points (4, 12, 24 and 48 h after infection) were then sorted for single cell transcriptomic analysis with an adapted Smart-seq2 protocol (Zanini et al., 2018). The DENV data set comprises 933 infected cells (MOI = 1 or 10) and 303 controls (MOI = 0), while the ZIKV data set is composed of 488 infected cells (MOI = 1) and 403 controls. Before submitting the analysis to the webCEMiTool platform, both data sets were log10 transformed and genes that were not expressed in more than 80% of the samples were removed. The data sets were then split by virus and by time point and used as input (“Expression file” field) to webCEMiTool. In addition to the gene expression data, we also provided to webCEMiTool the sample phenotypes (i.e., viral loads) and Reactome gene sets.

**Figure 2:**
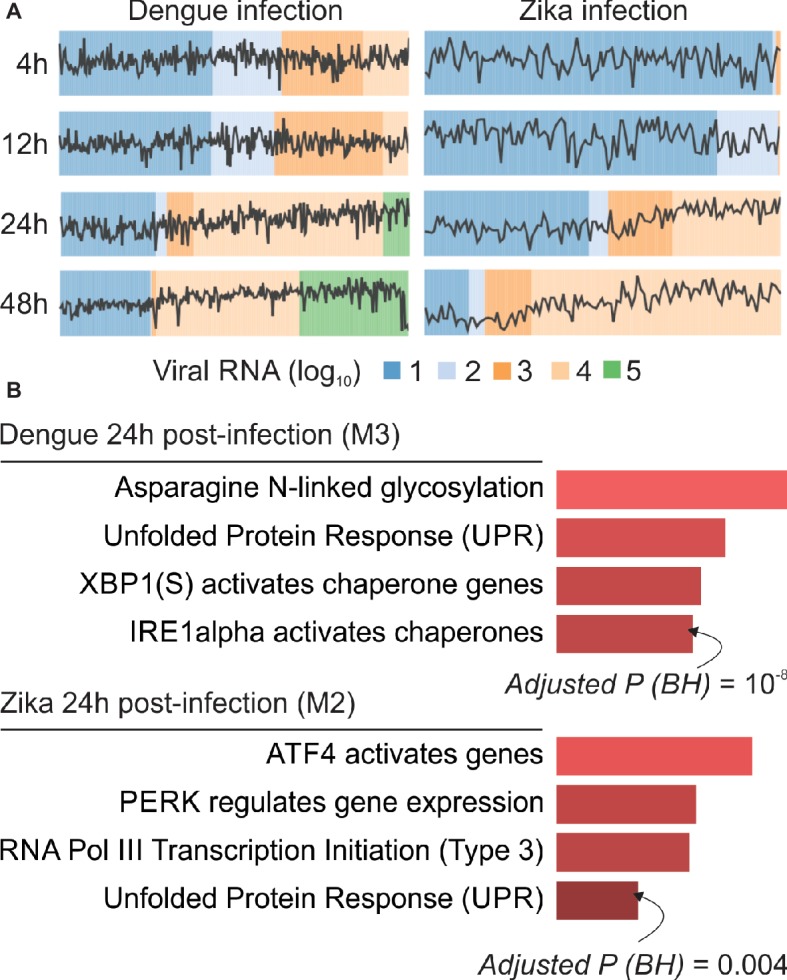
webCEMiTool applied to single-cell RNA-seq data. **(A)** Profile plot of co-expressed gene modules. We selected one representative module for each time point post-dengue virus infection (left) or post-Zika virus infection (right). The black line represents the median expression activity of genes from the modules across each sample. The colors represent the different amount of virus RNA within the cell. **(B)** Overrepresentation analysis of selected modules at 24 h post-virus infection. The bar graphs were adapted from the Enrichr webtool linked to webCEMiTool. The bars are proportional to the −log10 adjusted p-value (Benjamini-Hochberg) of the enriched pathways in a module.

Our webCEMiTool analyses generated an average of six modules per time point in DENV infection and more than eight modules per time point in ZIKV infection. We have selected one module per time point as a representative of our findings (Figure 2A). It is clear that at 24 and 48 h post-infection, the expression activity of representative modules increases according to the viral load (Figure 2A). We next performed the pathway enrichment analysis of the representative modules at 24 h post-infection using the webCEMiTool link for Enrichr (Figure 2B). These findings not only corroborate what was described in the original publication (Zanini et al., 2018) but also provide new insights about the physiopathology of dengue and Zika virus infections.

## Discussion

Although few similar web-based applications were developed to perform co-expression gene analysis (Tzfadia et al., 2016; Desai et al., 2017), these tools do not provide comparable results to webCEMiTool. One such application is GeNET (Desai et al., 2017). This webtool was designed to facilitate gene co-expression analyses and provides enrichment analysis and gene-to-gene networks. However, it only performs these analyses for three organisms (*R. capsulatus*, *M. tuberculosis*, and *O. sativa*). Another example is CoExpNetViz (Tzfadia et al., 2016), a webtool designed for the visualization and construction of gene networks. Similar to GeNET, CoExpNetViz is somewhat limited with respect to the organisms as it is stated to be primarily designed for plant transcriptomes. The webCEMiTool aims to provide co-expression analyses for any organism. Moreover, although CoExpNetViz is presented as a web-based application, its results are returned to users as a compressed folder containing a README.txt file with instructions on how to visualize their results on the Cytoscape app. The users have then to manually insert into Cytoscape the several different output files provided by the tool. These additional steps can also make the process error-prone and possibly daunting to users unfamiliar with Cytoscape. The webCEMiTool offers much more convenient browser-displayed results.

We also showed that webCEMitool is able to analyze single-cell RNA-seq data faster and efficiently. Our results returned relevant information about the biological processes involved with dengue and Zika virus infection. All this analysis were performed in an automated and practical manner, with no need for the user to have deep understanding on the internal processing of gene co-expression data analysis.

## Author Contributions

LC, PR, BG-C, and MA-P performed the analyses. LC, GS-H, and VM-C developed the webtool. HN conceived the tool and supervised the work. All authors help in the writing of the paper.

### Conflict of Interest Statement

The authors declare that the research was conducted in the absence of any commercial or financial relationships that could be construed as a potential conflict of interest.

## Footnotes

^2^https://cemitool.sysbio.tools/tutorial
